# D1 and D2 Inhibitions of the Soleus H-Reflex Are Differentially Modulated during Plantarflexion Force and Position Tasks

**DOI:** 10.1371/journal.pone.0143862

**Published:** 2015-11-24

**Authors:** Fernando Henrique Magalhães, Leonardo Abdala Elias, Cristiano Rocha da Silva, Felipe Fava de Lima, Diana Rezende de Toledo, André Fabio Kohn

**Affiliations:** 1 School of Arts, Sciences and Humanities, Universidade de São Paulo, EACH-USP, São Paulo, SP, Brazil; 2 Biomedical Engineering Laboratory, Universidade de São Paulo, EPUSP, Avenida Professor Luciano Gualberto, Travessa 3, n.158, São Paulo, SP, Brazil; 3 Neuroscience Program, Universidade de São Paulo, São Paulo, SP, Brazil; 4 Department of Biomedical Engineering, School of Electrical and Computer Engineering, University of Campinas, Campinas, SP, Brazil; University of Ottawa, CANADA

## Abstract

Presynaptic inhibition (PSI) has been shown to modulate several neuronal pathways of functional relevance by selectively gating the connections between sensory inputs and spinal motoneurons, thereby regulating the contribution of the stretch reflex circuitry to the ongoing motor activity. In this study, we investigated whether a differential regulation of Ia afferent inflow by PSI may be associated with the performance of two types of plantarflexion sensoriomotor tasks. The subjects (in a seated position) controlled either: 1) the force level exerted by the foot against a rigid restraint (force task, FT); or 2) the angular position of the ankle when sustaining inertial loads (position task, PT) that required the same level of muscle activation observed in FT. Subjects were instructed to maintain their force/position at target levels set at ~10% of maximum isometric voluntary contraction for FT and 90° for PT, while visual feedback of the corresponding force/position signals were provided. Unconditioned H-reflexes (i.e. control reflexes) and H-reflexes conditioned by electrical pulses applied to the common peroneal nerve with conditioning-to-test intervals of 21 ms and 100 ms (corresponding to D1 and D2 inhibitions, respectively) were evoked in a random fashion. A significant main effect for the type of the motor task (FT *vs* PT) (p = 0.005, η^2^_p_ = 0.603) indicated that PTs were undertaken with lower levels of Ia PSI converging onto the soleus motoneuron pool. Additionally, a significant interaction between the type of inhibition (D1 *vs* D2) and the type of motor task (FT *vs* PT) (p = 0.038, η^2^_p_ = 0.395) indicated that D1 inhibition was associated with a significant reduction in PSI levels from TF to TP (p = 0.001, η^2^_p_ = 0.731), whereas no significant difference between the tasks was observed for D2 inhibition (p = 0.078, η^2^_p_ = 0.305). These results suggest that D1 and D2 inhibitions of the soleus H-reflex are differentially modulated during the performance of plantarflexion FT and PT. The reduced level of ongoing PSI during PT suggests that, in comparison to FT, there is a larger reliance on inputs from muscle spindles primary afferents when the neuromuscular system is required to maintain position-controlled plantarflexion contractions.

## Introduction

Presynaptic inhibition (PSI) is ubiquitous in the vertebrate spinal cord [1] and has been shown to modulate several neuronal pathways of functional relevance [2], even though the neural circuitry behind PSI remains elusive. Studies in humans have indicated that PSI levels may be influenced by descending inputs, by Ia muscle afferents and by spinal cord interneurons (which can, for example, be activated by cutaneous receptors, Golgi tendon organs, spindle secondary afferents) [3]. Probably the most studied effect of PSI is on Ia terminals originating from muscle spindles, due to its important role in regulating the stretch reflex circuitry and consequently the ongoing motor activity. Therefore, PSI of Ia terminals has been investigated in upper and lower limb muscles by applying a conditioning electrical stimulus (assumed to activate interneurons yielding primary afferent depolarization) to the nerve of the antagonist muscle and a test stimulus to the nerve of the agonist muscle with a conditioning-to-test interval of appropriate latency [4,5]. Due to the inhibitory effect associated with the PSI circuitry, the Hoffmann reflex (H-reflex) conditioned by the antagonist nerve stimulation will have a lower amplitude as compared to the H-reflex elicited without conditioning. Specific conditioning-to-test intervals (for reviews, see [4,6,7]) have been used in the literature, with the corresponding inhibitory effects being labeled as D1 inhibition or D2 inhibition, as will be mentioned ahead. Using the aforementioned technique, the effects of PSI have been shown to depend on the type of sensorimotor task being performed [8–11].

More specifically, two types of motor tasks (named force task, FT, and position task, PT) have been used in laboratory settings to investigate several mechanisms underlying motor control and performance [12–15]. The first task (FT) requires the subject to push against a rigid restraint and to match a submaximal target force by performing an isometric contraction. The second task (PT) requires the subject to keep the joint at a given position while supporting an inertial load by performing an anisometric contraction. Based on visual feedback of target force or position, the subject is instructed to “maintain force” during the isometric contraction (FT) and to “maintain position” in the anisometric contraction (PT) [12]. A recent study [16] reported differential cortical contributions (as evidenced by increased coherence between different regions of the brain) during a knee extension FT as compared to a PT, which might be associated with the variety of neurophysiological differences that have been reported between FTs and PTs. For instance, it has been shown that the recruitment order of motor units is usually similar for FTs and PTs [17,18], whereas the recruitment threshold and modulation of discharge rate depend on the type of motor task [19–21]. Baudry and colleagues [9–11], focusing on D1 inhibition (i.e. conditioning-to-test intervals of ~15ms [22]), showed that the larger amplitude of H-reflexes in the extensor and flexor carpi radialis muscles observed during PTs (as compared to FTs) [23,24] was associated with a differential presynaptic modulation of Ia afferent input between the two tasks (i.e. reduced PSI for PT as compared to FT).

The present manuscript extends the findings above by assessing the regulation of Ia afferent inflow by PSI during FTs and PTs of plantarflexion contractions. Investigating the regulation of Ia PSI converging onto the motoneuron pool of ankle muscles during FTs and PTs may provide useful information for better understanding the neuronal mechanisms associated with torque control at the ankle joint [8,25]. Several aspects related to central and peripheral mechanisms associated with the neuromuscular control of steady contractions are known to vary according to the muscle studied [26]. As PSI can be modulated by either peripheral or descending commands [27,28], it is not easy to predict whether Ia afferents from plantarflexor muscles may or may not show a task-dependent modulation of PSI as that reported for hand muscles by Baudry and colleagues [9–11]. For example, the soleus motoneuron pool receives weaker corticospinal projections compared to upper limb muscles such as hand and finger extensors [29,30]. Thus, the soleus muscle may have distinct PSI modulation during FTs *vs* PTs when compared to the upper extremity muscles investigated by Baudry and colleagues [9–11]. In the present study, we hypothesized that plantarflexion PTs, in comparison to FTs, should occur with lower levels of Ia PSI on the soleus motoneuron pool, as strengthened connections between afferent fibers from muscle spindles and motoneurons (by reduced Ia PSI) would be beneficial for controlling the steadiness of ankle position. On the other hand, as the position of the ankle is held constant during FTs, the control of the steadiness of ankle isometric forces (during FTs) should rely more heavily on sources of sensory feedback other than afferent information from muscle spindles.

Mizuno et al. [31] defined two phases of inhibition in response to conditioning stimulation, one named D1 occurring at conditioning-to-test intervals between 5 and 50 ms and a second one with conditioning-to-test intervals between 70 and 200 ms called D2. The D2 inhibition has been used in a variety of protocols to induce PSI, arguably without the interference of postsynaptic effects [32–37]. Therefore, the investigation of both D1 and D2 inhibitory responses has the potential to unravel differential mechanisms due to the different onset latency and time course of each inhibitory response. As both conditioning-to-test intervals corresponding to D1 and D2 inhibitions have been associated with a PSI mechanism [32–34,36,38,39], in the present study we expected a larger inhibitory effect (i.e. PSI) on reflex responses obtained during FTs as compared to PTs, regardless of the conditioning-to-test interval (D1 or D2) used to probe the PSI pathway.

The present study also included complementary experiments to explore whether the effect of D1 and D2 inhibitions during FT and PT could indeed be ascribed to a PSI mechanism or, alternatively, whether a change in the monosynaptic reflex excitability could determine the effects of D1 and D2 conditioning independent of the ongoing levels of Ia PSI ([31], see Discussion for details). More specifically, it was hypothesized that the amount of heteronymous Ia facilitation of the soleus H-reflex induced by conditioning stimuli applied to the femoral nerve would be increased in the task (i.e. PT or FT) associated with the lower levels of D1 and D2 inhibitions [29].

Therefore, the aim of this study was to investigate the influence of the type of the task (i.e. FTs *vs* PTs) on the modulation of Ia presynaptic inhibition of the soleus muscle afferents. Both D1 and D2 inhibitions (with conditioning-to-test intervals around 20 ms and around 100 ms [31], respectively) were investigated, so as to establish comparisons between different methods that have been employed to probe spinal pathways associated with PSI of the soleus Ia terminals. Complementary experiments explored the heteronymous Ia facilitation of the soleus H-reflex induced by conditioning stimuli applied to the femoral nerve in order to obtain additional evidence on the presynaptic origin associated with the results of D1 and D2 inhibitions.

## Materials and Methods

### Participants

Ten subjects (5 males, 5 females, 27.9 ± 7.8 (SD) age) volunteered to participate in this study. All subjects were healthy and physically active, with no known musculoskeletal injuries or neurological disorders. All were right-footed. The experiments were conducted according to the Declaration of Helsinki and all procedures were approved by the Human Ethics Committee of the Institute of Biomedical Sciences at the University of São Paulo. Each subject signed an informed consent document prior to the experimental sessions.

### EMG Acquisition and Nerve Stimulation

The electromyograms (EMGs) were recorded differentially using round-shaped surface electrodes (Ag-AgCl, 0.8 cm diameter, with an inter-electrode distance of 2 cm) over the right soleus, medial gastrocnemius, lateral gastrocnemius, tibialis anterior, vastus lateralis and semitendinosus muscles. The electrodes were positioned at standard SENIAM positions, except for the soleus: the most proximal contact was 4 cm beneath the inferior margin of the two heads of the gastrocnemii muscles. A ground electrode was placed over the tibia (left leg). The EMG signals were amplified and filtered (5 Hz to 2 kHz) by a MEB-2300K system (Nihon-Kohden, Japan) and sent to an A/D interface (National Instruments, Austin, TX.) with a 5-kHz sampling rate. Data were stored on hard disk for later off-line processing.

Direct muscle responses (M-waves) and H-reflexes from soleus were obtained by electrical stimulation (rectangular pulses, 1ms duration, generated by the MEB-2300K) applied to the posterior tibial nerve (test stimuli) delivered through surface electrodes (area = 2 cm^2^) positioned in the popliteal fossa. For D1 and D2 studies, conditioning stimuli were applied to the common peroneal nerve at the fibular head. Conditioning stimulus intensity was set at 1.1 times the motor threshold, with motor threshold corresponding to the smallest observable M-wave of the tibialis anterior muscle. A custom-written program in LabView (National Instruments, USA) controlled the delivery of trigger pulses to the stimulator according to the conditions described ahead.

### Experimental Setup

**Fig 1:**
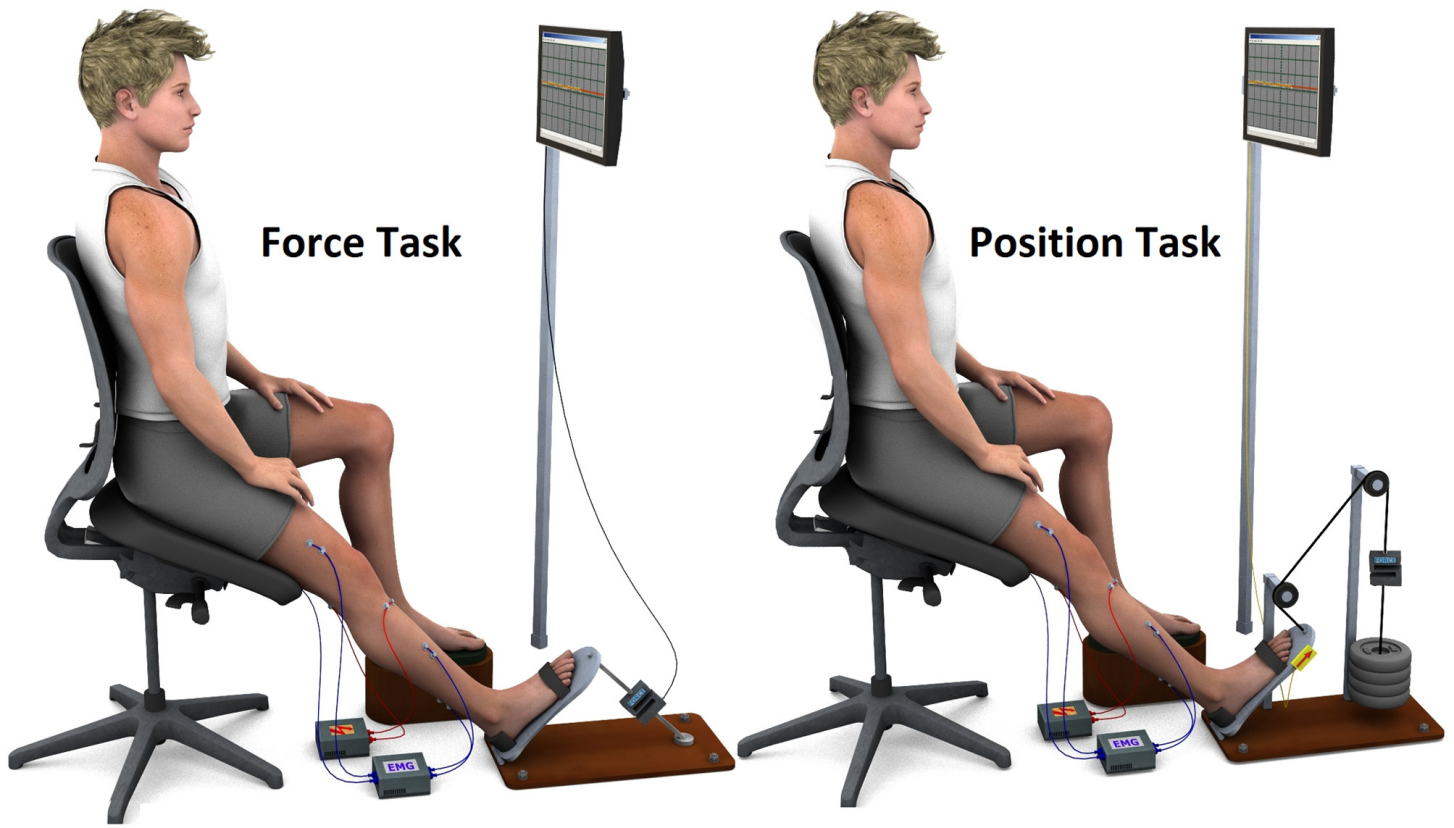
Schematic representation of the experimental setup. Subject positioned during the force (left part) and position tasks (right part). In the force task (FT), the right foot was strapped to a pedal connected to a force transducer and the subjects had the ongoing plantarflexion force as visual feedback (signals from the force transducer). In the position task (PT), the pedal was attached to the load via a pulley system and visual feedback of the ankle angle (signals from the accelerometer, depicted at the right side of the base of the pedal) was provided. The scheme shows the location of recording and stimulation electrodes (connected with blue and red wires, respectively) for posterior tibial nerve and common peroneal nerve stimulation and for soleus, tibialis anterior, semitendinosus, and vastus lateralis EMG acquisition.

Subjects were seated comfortably on a customized chair, with armrest and headrest, designed for measuring ankle torque during isolated isometric plantarflexion contractions (i.e. during FT) and for measuring ankle torque and position during anisometric plantarflexion contractions (i.e. during PT). In FT, the subject’s right foot was firmly strapped (with velcro placed over a thin foam pad, without causing discomfort) to a rigid pedal connected to a force transducer (Transtec N320, Brazil). In PT, the pedal was attached to the load via a pulley system and a force transducer was adequately positioned for torque measurements (see the scheme in Fig 1). Ankle angle (during PT) was measured with an accelerometer (ADXL103CE, Analog Devices) that was taped to the lateral side of the pedal to which the subject’s right foot was strapped. For both FT and PT conditions, the ankle of the right leg was maintained at 90°, the knee was fully extended (180°) and the hip was at approximately 120°. During PT, visual feedback of the ankle angle was provided to the subjects on an LCD monitor placed at eyes level. The distance from the subjects to the LCD monitor was maintained constant during the experiments and was similar for all subjects. During FT, subjects had the ongoing plantarflexion torque as visual feedback. A custom-written program in LabView (National Instruments, USA) provided the visual feedback (i.e. target force or position) as a horizontal line in the middle of the monitor and the force or position exerted by the subjects as a thinner line progressing with time from left to right. Additionally, the same LabView program provided the experimenter with an estimation of the ongoing RMS level of the plantarflexors EMGs, (based on moving windows of 100 ms duration).

## Procedures

At the beginning of the session, subjects were asked to perform two maximal isometric voluntary contractions (in FT) of plantarflexion. Trials of maximal isometric voluntary contractions lasted 3–4 s each and had verbal encouragement and visual feedback of the exerted force, with 2-min rest between each trial. The maximum force value achieved was taken as the maximal voluntary contraction force value. The target torque level for the force-matching tasks (i.e. during FT) was set at ~10% of the subjects’ maximal voluntary contraction force. This value was chosen because it corresponds approximately to the plantar flexion torque exerted during quiet standing [40,41].

Thereafter, subjects performed familiarization trials in both conditions (three trials in FT and then three trials on PT). No nerve stimulation was delivered in the familiarization trials. Subjects were instructed to maintain their torque/position on the target as accurately and as consistently as possible for 57 s. During the FT, subjects tried to keep the exerted isometric plantarflexion torque amplitude at target level (~10% of maximal voluntary contraction) and visual gain was adjusted so that the screen of the LCD monitor covered a range equivalent to 2% of the maximal voluntary contraction (i.e. 1% of the maximal voluntary contraction below and above the target force). For the PT, subjects were instructed to keep the ankle joint as close as possible to 90°. The experimenter adjusted the load (weight plates) so that the subjects, when keeping their ankle angle as constant and accurate as possible, had the soleus EMG level (as measured by RMS values) similar to the one measured during the FT. During PT, visual gain was adjusted so as to match the subject’s performance during FT. More specifically, during the familiarization PT trials, the experimenter adjusted the visual gain so that the screen area covered by angle fluctuations (measured during PTs) approximately matched the one covered by torque fluctuations during FTs.

After the familiarization trials, the maximal motor wave (Mmax) amplitude was estimated for each subject. At least five Mmax were elicited at 0.2 Hz by delivering supramaximal electrical stimuli to the posterior tibial nerve, while the subjects exerted a force-matching task at the target level (i.e. FT, at ~10% of the maximal voluntary contraction). The experimenter progressively increased stimulus intensity until a constant, maximal M wave was observed. The peak-to-peak amplitude of the Mmax was estimated online from single EMG sweeps displayed on the screen of an oscilloscope (Hewlett-Packard, Colorado Springs, CO). The Mmax value was defined as the maximal peak-to-peak amplitude observed among the Mmax measurements.

Lastly, subjects performed FTs and PTs (as described above) while a stimulation paradigm was delivered to assess the modulation of D1 and D2 inhibitions of the soleus H-reflex. During these experiments, H-reflexes of the soleus muscle were elicited at 0.25 Hz, so that the subjects had enough time (i.e. 4 s) to return to the target force/position level after the perturbation caused by the generation of an H-reflex. Reflex amplitudes were estimated online from single EMG sweeps displayed on the oscilloscope. At the beginning of each trial (in FT and PT), five unconditioned H-reflexes were delivered. These reflex responses allowed the experimenter to adjust the intensity of the test stimuli so that the amplitude of the unconditioned reflex (i.e. control reflex, monitored by the oscilloscope) corresponded to ~20% of the amplitude of the Mmax (these five initial responses were discarded from the analyses). Then, control (unconditioned) H-reflexes and H-reflexes conditioned by a single electrical pulse (1 ms duration) applied to the common peroneal nerve (1.1 x motor threshold) at conditioning-to-test intervals of 21 ms or 100 ms (corresponding to D1 and D2 inhibitions, respectively) were evoked in a random fashion. Therefore, three valid reflex responses for each condition (control, D1, and D2) were obtained during the 57-s tasks (FT and PT). Subjects performed seven trials in FT and seven trials in PT (which resulted in a sample of 21 reflexes for each condition). The order of the tasks was counterbalanced across subjects.

The EMG levels of the tibialis anterior, vastus lateralis and semitendinosus muscles were monitored online to ensure that no significant levels of activation occurred in these muscles. If a significant level of activation was detected (as defined by RMS values two times higher than that observed in rest), the subject was instructed to avoid contraction of the specific muscle and the trial was rejected and repeated. Additionally, the experimenter visually checked the background RMS values of the EMG signals from medial gastrocnemius and lateral gastrocnemius muscles to ensure that the activation levels of these muscles did not change considerably between conditions (FT *vs* PT), as it might had been the case if, for example, the subjects have used different mechanical strategies to press the foot against the pedal. Moreover, the M-wave in the EMG of the tibialis anterior muscle was constantly monitored to ensure a maintained efficacy of the conditioning stimulation throughout the experiment. No differences in the amplitude of the tibialis anterior M-wave were detected among conditions.

### Data Analysis

In order to evaluate the effect of task type (FT and PT) on both D1 and D2 inhibitions, the amount of inhibition was measured for each conditioning protocol [37,42]. Therefore, conditioned H-reflexes were expressed as the amount of inhibition, which consisted of the difference between control and conditioned reflex responses over its control value, as follows:

Amount of Inhibition = [(mean control - mean conditioned) / mean control)]  * 100%

in which the term “mean conditioned” refers to the averaged peak-to-peak amplitude of the reflex responses that were conditioned by electrical pulses applied to the common peroneal nerve for either D1 (conditioning-to-test interval = 21 ms) or D2 (conditioning-to-test interval = 100 ms) inhibitions. Thus, the amount of inhibition for FT and PT conditions were calculated for each subject. A two-way ANOVA with repeated measures [type of contraction (“FT” *vs* “PT”) and duration of conditioning-to-test interval (D1 *vs* D2)] was used to detect main effects and interactions among experimental conditions. As a significant interaction was detected, simple effects tests (with Bonferroni’s correction) were conducted for the contrasts of theoretical interest. Additionally, confidence intervals (CIs) in conjunction with graphical presentation were used to allow appropriate data interpretation [43,44]. More specifically, the mean amount of inhibition for each condition and the mean difference in the amount of inhibition between FT and PT conditions (with their respective within-subjects CIs) were used to evaluate the pattern of differences between conditions.

As the measurements of amount of inhibition described above may eventually “hide” information on difference among control parameters (i.e. unconditioned reflexes), a t-test for paired samples was used to compare the amplitude of the control (unconditioned) H-reflexes measured during FT and PT trials. Similarly, paired t-tests were used to compare the levels of background soleus EMG (as measured by RMS values computed after removing the EMG signals corresponding to stimulus artifacts, M-waves, H-reflexes and silent periods) between FT and PT trials.

Statistical analysis were performed using the statistical package SPSS 15.0 for Windows (SPSS, Inc., Chicago, IL), with significance level set at p < 0.05. Effect sizes are reported using partial eta-squared (η^2^_p_) indices [45], which indicate the percent of variance in each of the effects (or interaction) that is accounted for by that effect (or interaction). Within-subjects CIs (95% CIs) were computed as described in [43,46].

### Complementary Experiments

An additional set of experiments was performed in three subjects. They had previously participated in the main experiments described above and showed stronger D1 and D2 inhibitions during FT as compared to PT.

The protocol employed in the additional experiments replicated the previous one described above, except that the trials (in FT and PT conditions) consisted in evoking control H-reflexes (unconditioned), and H-reflexes conditioned by a stimulus (single rectangular pulse, 1 ms duration, intensity of 0.8 x motor threshold) applied to the femoral nerve. Conditioning stimuli were delivered with the active electrode (round shaped, area = 2 cm^2^) positioned over the femoral triangle and the reference electrode (rectangular, area = 20 cm^2^) over the posterior and upper aspect of the thigh. The interval between conditioning and test stimuli was chosen based on a previous exploration of the time course variation of the heteronymous Ia facilitation for each subject (not recorded) and ranged between -3 and -4 ms.

Descriptive statistics were used to establish comparisons between the amplitude of H-reflexes conditioned by heteronymous Ia facilitation and the amplitude of control H-reflexes for each experimental condition (i.e. during FT and PT), with the amplitude of the reflexes expressed as a percentage of the mean amplitude observed in the control condition.

## Results

**Fig 2:**
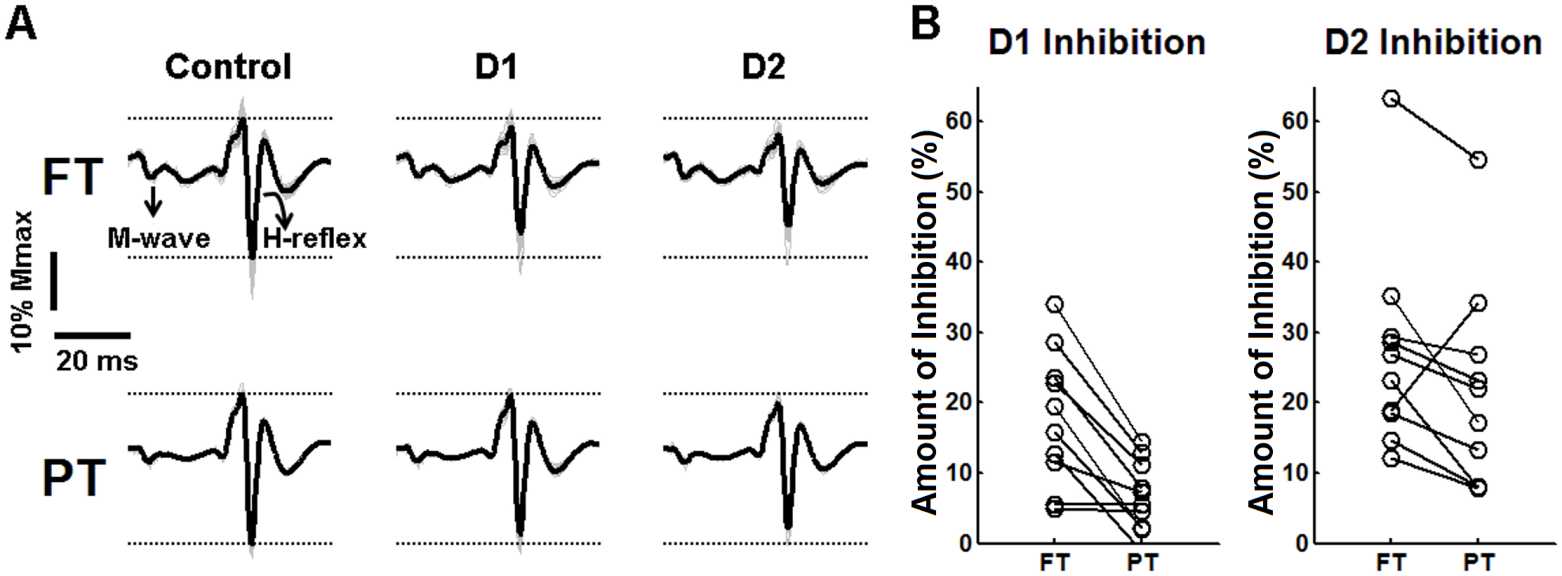
Effects of D1 and D2 inhibitions on soleus H-reflex amplitude during plantarflexion force and position tasks. (A) Representative data for one subject, showing raw H-reflexes (thin lines) obtained during FT (upper traces) and PT (lower traces) trials. M-waves and H-reflexes are shown, as indicated by the arrows in the upper signal. The thick lines indicate the average M-waves and H-reflexes. Three experimental conditions are depicted: control condition (left traces), D1 inhibition (middle traces), and D2 inhibition (right traces). (B) Individual values of the amount of inhibition computed for D1 and D2 conditions during force and positions tasks (FT and PT).

Fig 2A shows representative recordings of raw control and conditioned soleus H-reflexes from a single subject, illustrating changes in H-reflex amplitudes for the different conditions (i.e. under control, D1 and D2 inhibition, shown in the left, middle and right columns, respectively). It is noteworthy that this subject showed reduced H-reflex amplitudes under D1 and D2 inhibitions as compared to the control condition (compare the peak-to-peak amplitudes of the averaged H-reflexes in D1 and D2 with those of the control responses, as indicated by the dashed horizontal lines). Moreover, the stronger effect of D1 and D2 inhibitions during FT trials as compared to PT trials is evident for this subject (compare the reductions in H-reflexes associated with D1 and D2 inhibitions between upper traces and lower traces). Fig 2B depicts individual values of the amount of inhibition induced by D1 and D2 conditioning, during FT and PT trials. For both D1 and D2 inhibitions, 9 out of 10 subjects showed higher levels of amount of inhibition during FT as compared to PT.

**Fig 3:**
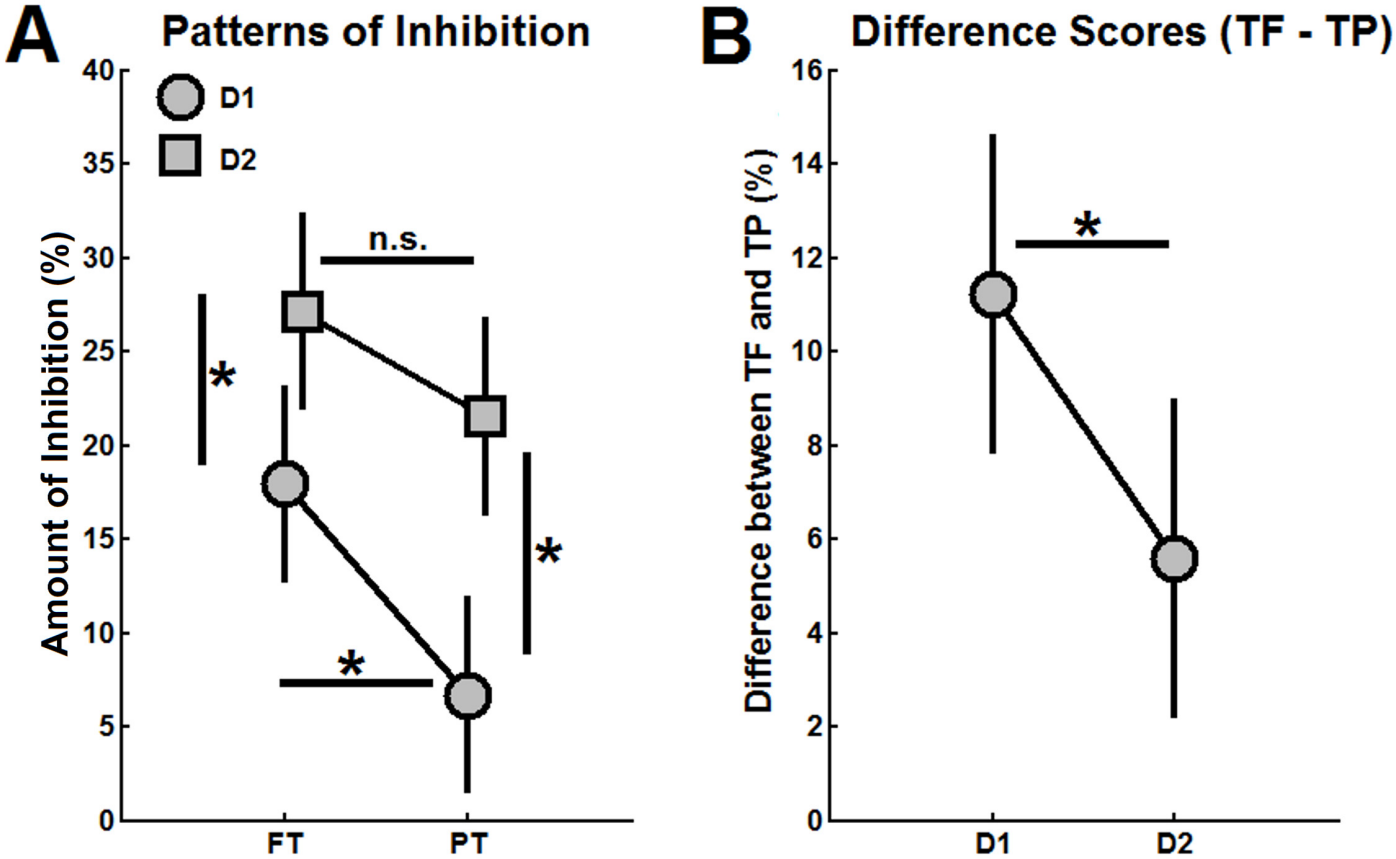
Condition means and interaction plot with 95% within-subjects CIs. (A) Average values of the amount of inhibition computed for D1 and D2 conditions during force and positions tasks (FT and PT), with 95% within-subjects CIs represented as error bars. B) Mean differences in the amount of inhibition between FT and PT conditions (error bars show the associated within-subjects 95% CIs). Asterisks indicate significant differences (p < 0.05) between conditions, as revealed by two-way ANOVA and simple effects tests. Not significant = “n.s.”

The two-way ANOVA test performed on amount of inhibition measures revealed a significant main effect for the “type of contraction” (Wilks’ Lambda = 0.397, F_(1,9)_ = 13.64, p = 0.005, η^2^_p_ = 0.603), indicating higher inhibition for the FT as compared to the PT condition. A significant main effect was also obtained for the factor “duration of conditioning-to-test interval” (Wilks’ Lambda = 0.430, F_(1,9)_ = 11.909, p = 0.007, η^2^_p_ = 0.570), with higher inhibition observed for D2 as compared to D1. ANOVA also revealed a significant interaction between the factors “type of contraction” and “duration of conditioning-to-test interval” (Wilks’ Lambda = 0.605, F_(1,9)_ = 5.868, p = 0.038, η^2^_p_ = 0.395). Simple effects tests indicated that the amount of inhibition was higher for D2 inhibition (as compared to D1) in both TF (p = 0.032, η^2^_p_ = 0.417) and TP (p = 0.003, η^2^_p_ = 0.639) conditions. Comparisons between the “type of contraction” showed that the amount of inhibition in FT was higher than in PT for D1 inhibition (p = 0.001, η^2^_p_ = 0.731), whereas no significant difference between FT and PT was found for D2 inhibition (p = 0.078, η^2^_p_ = 0.305). The panels in Fig 3 allow a graphically based interpretation of these results [43]. Fig 3A shows the average values of amount of inhibition with 95% within-subjects CIs depicted as error bars, illustrating the larger inhibitory effect of D2 as compared to D1 and the higher levels of inhibition in FT as compared to PT (with pronounced differences between FT and PT for D1, which is not evident for D2). The mean values of the individual differences in the amount of inhibition between FT and PT conditions (with the associated 95% within-subjects CIs) are shown in Fig 3B, indicating that the differences in the amount of inhibition (from FT to PT) were more prominent for D1 inhibition as compared to D2 inhibition.

**Fig 4:**
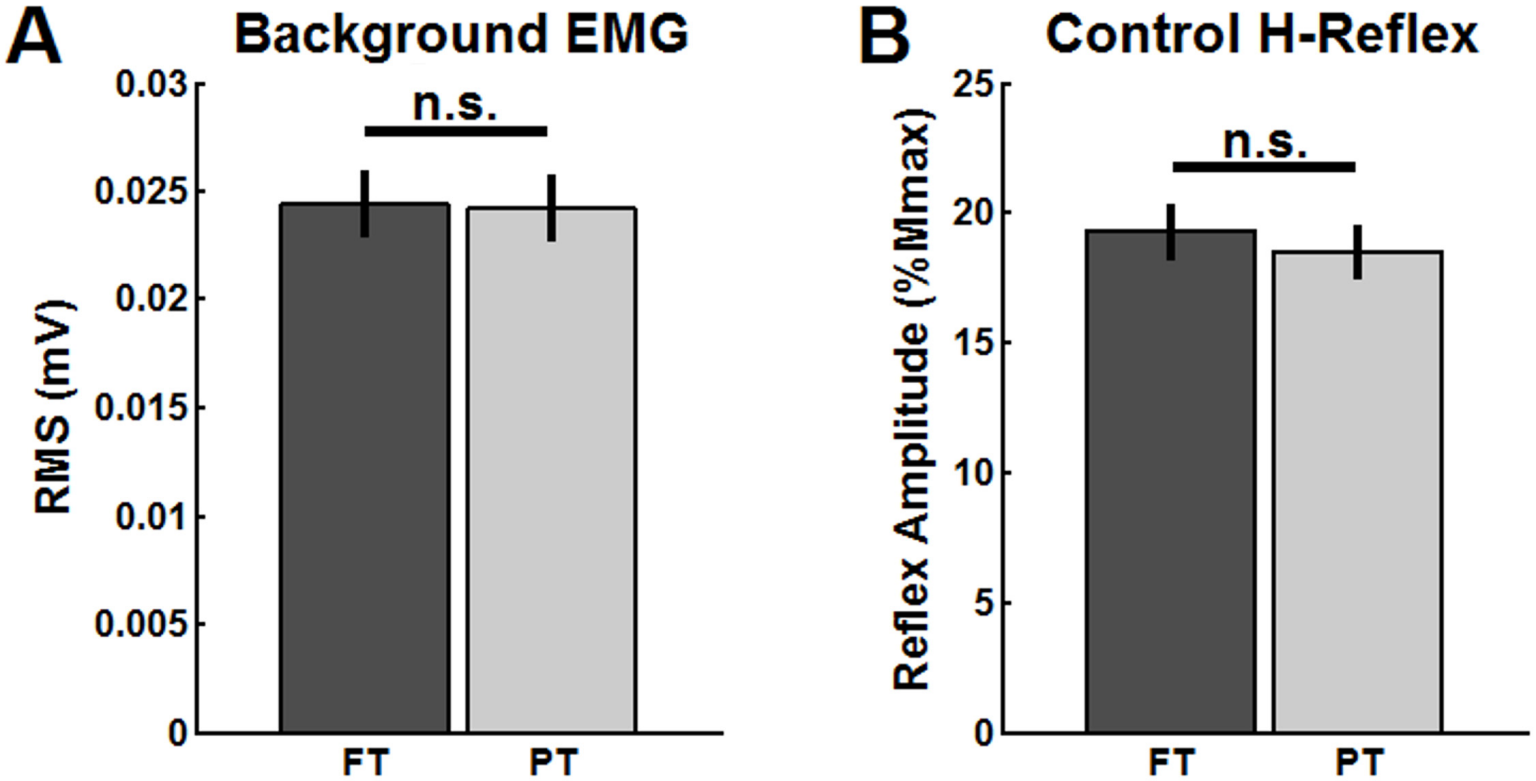
Amplitude of soleus EMG and control H-reflexes. (A) Average amplitude of soleus background EMG (expressed in mV) and (B) control H-reflexes (expressed in % of Mmax). Error bars depict within-subjects 95% CIs. There were no significant differences (n.s.) in soleus EMG activation and control reflex amplitudes between FT and PT trials (p>0.05).

Lastly, the t-tests performed on the levels of soleus EMG activation (RMS values) and on the amplitude of the control (unconditioned) H-reflexes revealed no significant differences between FT and PT trials (T_(9)_ = 0.29, p = 0.77, η^2^_p_ = 0.010 and T_(9)_ = 1.37, p = 0.20, η^2^_p_ = 0.172, for soleus EMG and control H-reflexes, respectively). These results indicate that the background levels of soleus EMG and the amplitude of control reflexes did not change considerably between FT and PT. Fig 4A and 4B show the averaged levels of background EMG (as measured by RMS values) and averaged amplitude of the control reflexes (expressed in %Mmax), respectively. Note that control H- reflexes were consistently maintained at ~20% Mmax during both FT and PT trials.

**Fig 5:**
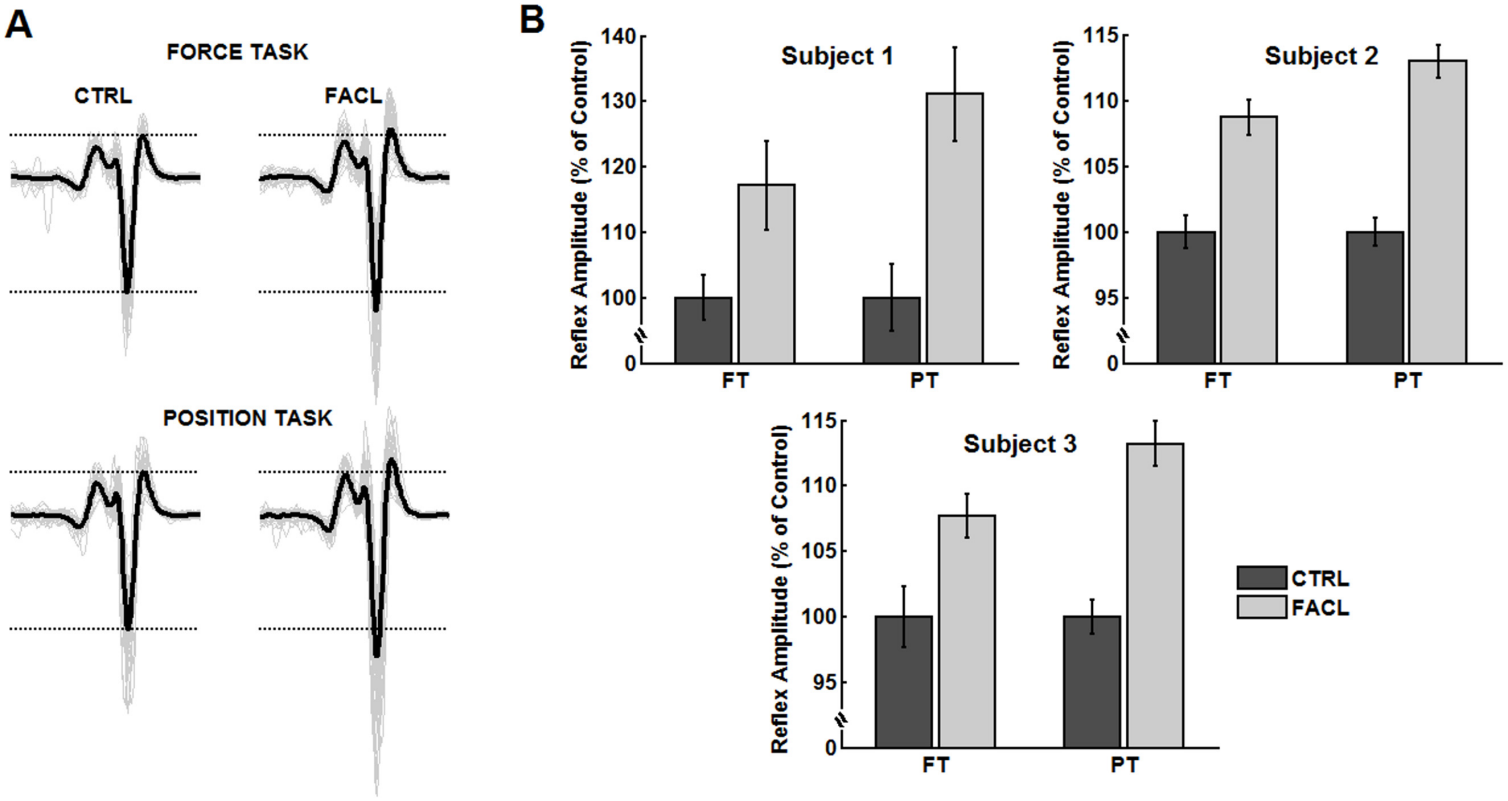
Heteronymous Ia facilitation from the quadriceps muscle on soleus H-reflex. (A) Representative data for one subject, showing raw EMG recordings with control H-reflexes (CTRL, left panels) and H-reflexes under heteronymous Ia facilitation (FACL, right panels), for both force task (upper panels) and position task (lower panels). Single reflexes are drawn in light gray (superimposed sweeps) and the averaged waves are drawn in thicker black lines. (B) Averaged H-reflex amplitudes (normalized by the mean amplitude of the control H-reflexes) computed for control (CTRL) and heteronymous Ia facilitation (FACL) conditions, for each subject. Note the larger heteronymous facilitation during PT than during FT.

The complementary experiments showed that the three subjects evaluated using the femoral nerve conditioning had a stronger heteronymous Ia facilitation during PT as compared to FT. Fig 5A shows representative recordings of control (unconditioned) H-reflexes and H-reflexes under heteronymous facilitation (i.e. femoral nerve stimulation), for both FT and PT conditions. During FT and PT, respectively, heteronymous facilitation increased the mean H-reflex amplitude (with respect to its control value) by 17.1% and 31.1% for Subject 1, by 8.7% and 13.0% for Subject 2 and by 7.6% and 13.2% for Subject 3. Fig 5B shows averaged H-reflex amplitudes (normalized by the mean amplitude of the control H-reflexes), illustrating the larger effect of heteronymous facilitation during PT than during FT.

## Discussion

The present results showed that soleus H-reflexes were more depressed by D1 and D2 inhibitions during FT than during PT (Figs 2B and 3A), with a parallel increase in heteronymous Ia facilitation in PT as compared to FT (Fig 5). Taken together, these results indicate that during plantarflexion PTs the neuromuscular control system sets lower levels of ongoing PSI on the soleus Ia afferents than during FTs. These results are lower limb counterparts of previous reports by Baudry and colleagues [9–11] on motor tasks performed by forearm muscles. They showed a differential modulation of D1 inhibition during FTs and PTs for contractions of the flexor and extensor carpi radialis muscles. It has been shown that the regulation of afferent inflow by PSI at Ia terminals may be modulated by either peripheral or descending commands [27,28], being altered according to the motor task [32] and even by mental activity [47]. Therefore, as commented in the Introduction, the present results could not have been predicted beforehand from the available knowledge on the forearm [9–11] due to the many putative mechanisms (of central and peripheral origin) that may act differentially on the spinal neural circuitry controlling lower limb muscles such as the soleus.

The modulation of Ia PSI during FT and PT trials was investigated by assessing the decrease of the soleus H-reflex following conditioning stimuli applied to the common peroneal nerve, with two different conditioning-to-test intervals (i.e. 21 and 100 ms, corresponding to D1 and D2 inhibitions, respectively) [31]. While there is no agreement on the better method to assess changes in the levels of PSI, conditioning-to-test intervals corresponding to D1 and D2 inhibitions have been used extensively to study the PSI circuitry [32–34,36,38,39]. One putative conjecture is that D1 and D2 are part of the same pathway, and a ‘facilitatory’ period between D1 and D2 could be ascribed to contamination from cutaneous inputs [37,48]. The present study showed a larger effect of D2 inhibition as compared to D1 inhibition (irrespective of the task being performed, see Fig 3A). Furthermore, D1 inhibition was associated with a stronger reduction in PSI levels from TF to TP in comparison to D2 inhibition (see Fig 3B), as evidenced by the higher difference scores (FT − PT) in the amount of inhibition for D1 as compared to D2 (Fig 3B). Therefore, it is suggested that D1 and D2 inhibitions may be conveyed by different subsets of interneurons that are differentially modulated during the performance of plantarflexion FTs and PTs.

Different methods have been proposed to estimate changes in presynaptic inhibition of Ia terminals during voluntary contraction in humans. However, none of these methods can provide by itself unequivocal evidence for a change in presynaptic inhibition of Ia terminals [49]. For example, the D1 and D2 methods reflect the excitability of interneurons mediating primary afferent depolarization, which can be influenced by an occlusion between a given central/peripheral input and the conditioning volley [49]. On the other hand, the reflex facilitation evoked by stimulation of heteronymous Ia fibers reflects the level of ongoing presynaptic inhibition of Ia afferents mediating the conditioning volley, but may be contaminated by changes in the recruitment gain of the reflex [50] and post-activation depression [51]. Therefore, the use of different methods has been advocated, with the rationale that plausible interpretations may be proposed if congruent results are obtained by the different methods [49]. Thus, in addition to the experiments with D1 and D2 inhibitions, we performed complementary experiments that assessed the levels of ongoing presynaptic inhibition exerted onto heteronymous Ia afferents from the quadriceps muscle to soleus motoneurons [52]. The preliminary results obtained from 3 subjects clearly suggested an enhanced heteronymous facilitation during PT as compared to FT (Fig 5), thereby reinforcing the evidence that the level of PSI is lower during PT as compared to FT.

In this study, we chose to control H-reflex amplitude by keeping unconditioned H-reflexes at ~20%Mmax (see Fig 4B), so as to presumably recruit the same motor units throughout the experiment (for a given condition). Besides producing control reflexes that are a constant in percentage [48], H-reflexes at ~20%Mmax are sensitive to both excitatory and inhibitory inputs and are usually on the rising edge of the recruitment curve [4]. For two subjects, H-reflexes at ~20%Mmax were accompanied by small M-waves, which further assured a constant stimulus intensity throughout the trials [53]. In these cases, M-waves were constant between control, D1 and D2 conditions; on the other hand they were slightly larger for FT as compared to PT trials (see example in Fig 2A). As the experimenter controlled the test stimulus intensity on the basis of the amplitude of unconditioned reflexes rather than on M-wave amplitude, increased stimulus intensity was necessary for FT as compared to PT, which is consistent with an increased level of PSI on soleus Ia afferents in FT.

In an investigation on the upper limbs of human subjects, Aimonetti et al. [54] showed that Ia afferents exciting low threshold motor units had a higher sensitivity to PSI than those exciting higher threshold motor units. In the present study, the effects of D1 and D2 inhibitions were investigated with H-reflexes of comparable sizes (i.e. control reflex with peak-to-peak amplitude ~20%Mmax, see Fig 4B). Thus, it is unlikely that the differential modulation of Ia PSI between FT and PT trials is associated with a mechanism dependent on the type of motoneurons recruited in the generation of H-reflex.

The present experiments were performed with matched levels of soleus EMG between FT and PT trials (Fig 4A). Additionally, no significant muscle activity occurred in the antagonist (tibialis anterior) and thigh muscles (semitendinosus and vastus lateralis) during the experiments. Therefore, we may exclude the possibility that different levels of coactivation and/or different levels of activity in adjacent muscles accounted for the differential modulation of Ia PSI between FT and PT trials.

Iles [33] showed that PSI of soleus Ia afferents was reduced after stimulation of cutaneous nerve branches (i.e. sural nerve) and also by lightly brushing the plantar surface of the foot. Therefore, cutaneous inputs from the foot exert important influences on the neural circuitry that generates PSI of soleus Ia afferents. In the present experiments, subjects performed FT and PT trials with similar cutaneous afferent activation, as the positioning of the foot and the velcro band used to strap the foot to the pedal did not change between FT and PT. Therefore, it is unlikely that differences in cutaneous input contributed to the enhanced D1 and D2 inhibitions found during FT as compared to PT trials.

It is also unlikely that Ib afferent input from Golgi tendon organs have contributed to the present results because muscle tension (and consequently Ib activation) was probably similar in FT and PT, as suggested by the lack of activation of tibialis anterior and thigh muscles and the matched levels of soleus EMG activation in both tasks (Fig 4A). On the other hand, it is difficult to predict whether different information from muscle spindles (type Ia and II afferent) may have played a role on the reduced levels of PSI observed during PT. Different levels of Ia afferent inflow from the antagonist (tibialis anterior) muscle (and perhaps from agonists) would certainly influence the levels of PSI at soleus Ia afferents. Although the position of the ankle was held constant during the experiments (around 90°), small ankle joint fluctuations occurred during PT and hence afferent information from muscle spindles might have been affected by ankle joint rotation. On the other hand, during FT (i.e. isometric task) the ankle position was held constant and hence afferent information from muscle spindles was modulated exclusively by muscle length variations associated with muscle activity and tendon compliance [55,56]. Therefore, negligible activation of tibialis anterior muscle spindles occurred during FT, which is not necessarily the case during PT (i.e. tibialis anterior spindle afferents might have been activated due to variations in ankle joint rotation). Additionally, afferent activity from the posterior muscle spindles was not necessarily similar between PT and FT, due to different relations between muscle length variation caused by ankle rotations and by muscle activation [55,57].

Different levels of PSI converging onto the Ia afferents acting on the soleus motoneuron pool must be counterbalanced by appropriate levels of descending drive to yield the similar levels of soleus EMG imposed for the FT and PT. However, whether an augmented descending drive is needed when the control system acts with higher levels of PSI (i.e. during FT), as suggested by Baudry et. al [9], may be difficult to postulate because afferent input from muscle spindles may not be the same in FT and PT, as commented above. In addition, Lauber et. al [58] showed that subthreshold transcranial magnetic stimulation suppressed the EMG of the first dorsal interosseus muscle to a greater extent when the subjects had visual feedback of the exerted position as compared to a condition with force feedback. One interpretation forwarded by the authors was that position-controlled muscular contractions might be associated with a greater motor cortical output as compared to force-controlled contractions. On the other hand, a recent study [16] showed evidence that the functional coupling between cortex activity and activation of knee extensor muscles (i.e. corticomuscular coherence) is similar during a PT as compared to a FT, whereas there is a greater involvement and communication between different regions of the brain (as evidenced by augmented cortico-cortical coherence) during the FT as compared to PT. Therefore, further studies are needed in order to better explore the differential cortical contributions between PT and FT and its functional relevance for force and position control.

A recent study [59] suggested that the statistical distribution of the pre-motoneuronal command spike trains (which includes both afferent inputs and descending drive) significantly influences plantar flexion torque variability as well as the synchronization of motor unit discharges. As the differential modulation of PSI during FT and PT affected the balance between descending and sensory inputs converging onto soleus motoneurons, it follows that plantar flexion contractions during FT and PT are probably driven by different pre-motoneuronal command statistics. This is in line with experimental results that showed a task-dependent modulation of motoneuronal discharge rate for the biceps brachii muscle [19–21].

Studies with a biologically plausible computational model [59–63] have suggested that the spinal cord anatomy and neurophysiology (e.g., motor unit types, synaptic connectivities, ordered recruitment), along with the modulation of afferent activity, may account for a variety of biomechanical and neurophysiological features associated with the control of the ankle joint during isolated plantarflexion contractions and during quiet stance. As the gain of afferent input from muscles spindles is dependent on the excitability of the primary afferent depolarization interneurons mediating PSI of Ia afferents, the reduced levels of ongoing PSI during PT suggests that, in comparison to the FT, there is a larger reliance on inputs from muscle spindles when the neuromuscular system is required to maintain position-controlled contractions.

A malfunction in some spinal pathways responsible for controlling the excitability of the stretch reflex has been associated with the development of spasticity in conditions such as following stroke and spinal cord injury. For instance, reduced PSI of Ia terminals [64–67] has been reported for these patients as compared to healthy controls. As weak steady contractions of spastic muscles may be used in the early stages of rehabilitation programs designed to recover/maintain function in these patients, the results of the present study suggest that it would be preferable to use protocols relying on isometric (FTs) rather than on anisometric (PTs) contractions, so as to favor recovery of lost spinal inhibition (specifically PSI) by training contractions under higher excitability of the primary afferent depolarization interneurons mediating PSI of Ia afferents. However, this conjecture is yet to be supported by future studies that explore short and long-term plastic changes in PSI circuitry in healthy and disease.

## Conclusion

The present study addressed the modulation of PSI as a function of the type of the plantarflexion task (force or position-controlled contractions). In comparison to FTs, PTs are undertaken with lower levels of Ia PSI converging onto the soleus motoneuron pool. Furthermore, it is suggested that D1 and D2 inhibitions of the soleus H-reflex are differentially modulated during the performance of plantarflexion force and position tasks. The results enhance our knowledge of the spinal physiology of plantarflexion neuromuscular control and may provide relevant implications for the practice of rehabilitation and training.
